# Communication in Autistic Adults: An Action-Focused Review

**DOI:** 10.1007/s11920-025-01616-6

**Published:** 2025-06-23

**Authors:** Ashley de Marchena, Nicole Cuneo, Emine Gurbuz, Maci Brown, James Trujillo, Jennifer Bergstrom

**Affiliations:** 1 AJ Drexel Autism Institute, Drexel University, 3020 Market Street, Suite 560, Philadelphia, PA 19104 USA; 2 Psychology Department, Princeton University, Princeton, New Jersey USA; 3 University of Portsmouth, Portsmouth, England; 4 Institute for Logic, Language & Computation, University of Amsterdam, Amsterdam, The Netherlands; 5 Department of Speech Language Pathology, Salus at Drexel University, Elkins Park, Pennsylvania USA

**Keywords:** Communication, Autism, Language, Nonverbal Communication, Disability, AAC

## Abstract

**Purpose of Review:**

This review focuses on recent research (primarily published between 2020–2024) describing unique aspects of communication in autistic adults. We review research on communication-related outcomes, as well as four communication topics frequently cited as important to autistic people (literal and non-literal language, augmentative and alternative communication, nonverbal communication, and double empathy).

**Recent Findings:**

A substantial proportion of autistic adults do not develop fluent language by adulthood. Autistic adults frequently experience communication barriers impacting relationships, employment, and health. These barriers arise from multiple sources (e.g., language, nonverbal communication, and unaccommodating environments and communication partners). Thus, it is essential to take a multifaceted approach to supporting autistic adults to ensure their communication success.

**Summary:**

We conclude that it is vital for clinicians and researchers to understand how autistic traits commonly manifest in adults and to accept and accommodate communication differences as they arise. To that end, we offer specific recommendations to help clinicians and researchers strengthen their interactions with the autistic people in their lives.

## Introduction

Effective communication – including both sending and receiving messages from others – is essential for human survival and quality of life. Broadly speaking, communication encompasses social behaviors, emotional responses, and cognitive and linguistic skills. When we use “communication” in this paper, we mean all modes of communication, not just spoken or signed language, including nonverbal communication such as gestures, facial expressions, body language, and the use of augmentative and alternative communication (AAC) systems. Autism spectrum disorder (hereafter, ‘autism’^1^) is characterized by differences and difficulties in “social communication” (described in more detail below); thus, a strong understanding of autistic communication is essential for clinicians and researchers working with this population.

### A Brief History of Communication in the DSM

Communication skills and behaviors are central to the clinical conceptualization of autism. Overall, the DSM autism criteria have evolved from viewing communication as a standalone domain to viewing it as inextricable from social interaction. The DSM-III [4] – the first edition to include autism as an independent diagnostic category in 1980 – emphasized gross language deficits and atypical speech patterns, such as echolalia and pronoun reversal. The DSM-III-R [5] expanded communication criteria to include verbal and nonverbal communication differences, including atypical eye contact and difficulty sustaining conversations. DSM-IV [6] and DSM-IV-TR [7] added delayed language development and repetitive language use. However, the most substantial change occurred with DSM-5 [8], in which communication and social interaction merged into a single domain, “Social Communication,” which requires persistent difficulties with social-emotional reciprocity, nonverbal communication, and relationships.

### Outcomes

Characterizing a “good” communication outcome is difficult to define and measure. However, by any definition, communication outcomes in autistic adults are highly heterogeneous.

#### Language and Communication Skills

Longitudinal studies of autistic adults followed since early childhood may provide the best data on communication outcomes, as they are less prone to sample biases that exclude participants with intellectual disabilities [9]. One proxy for language fluency is to examine rates of Autism Diagnostic Observation Schedule (ADOS; 10) module administration, as modules are tailored to examinees’ verbal status. Module 1 applies to examinees who do not use phrase speech and is thus indicative of minimally verbal status. According to recent results from longitudinal cohorts, 35% [10]—46% [11] of examinees received Module 1, demonstrating high rates of minimally-speaking status for adults diagnosed in childhood.

Beyond basic language fluency, as few as 25% of autistic adults diagnosed in childhood score in the average or above-average range when measuring communication as an adaptive behavior skill [11]. Even among adults with enough language fluency to self-report [11], our team has found high rates of positive screening for developmental language disorder (46%). Taken together, these studies suggest high rates of heterogeneity and high rates of communication disability among autistic adults.

#### Lived Experience

The outcomes described above rely primarily on outsider report or observation. But what about the opinions of autistic adults themselves? Communication involves both observable behavior (e.g., someone’s large vocabulary or expressive face, which can be noticed by others) and internal experiences (e.g., the subjective feeling of struggling to express one’s point, which can only be experienced by the communicator). Thus, it is vital to characterize the *experience* of communicating while autistic. Cummins and colleagues [12] asked autistic adults to report on their perspectives of communication, including communication preferences, perceived strengths and weaknesses, and desired support. Thematic analysis revealed that communication difficulties led to feelings of vulnerability and withdrawal from society.

#### Relationship to Quality of Life

Communication ability in adulthood, while an outcome in itself, also impacts quality of life and functional outcomes across domains. Building on outcome frameworks from the World Health Organization [13], Autistic Self Advocacy Network [14], and mothers of adults with Intellectual Disability [15], McCauley and colleagues [16] proposed a framework including autonomy, social relationships, and purpose, which could be measured across adults with higher and lower IQs. Verbal IQ was a strong and reliable predictor of positive outcomes, demonstrating the strong relationship between verbal reasoning and outcomes across the IQ spectrum. In a longitudinal study of 84 autistic adults, Friedman and colleagues [17] assessed conversational skills through open-ended interview questions. They found that vocabulary diversity, an indicator of structural language, was significantly associated with vocational independence: adults with a robust vocabulary were more likely to hold a job or advance their education. Additionally, maintaining conversation topics, a pragmatic language skill, was linked to friendships: effective social communication is crucial for establishing and maintaining social connections.

## The Current Review

The vast majority of autism research focuses on samples without significant cognitive (and thus, communication-related) disability [9], a major limitation of the field. In an effort to balance recent research and include varying levels of baseline communication abilities, we have focused our review on four essential topics: [1] literal and non-literal language, [2] augmentative and alternative communication (AAC), [3] nonverbal communication, and [4] allyship and the double empathy problem. With the exception of AAC – chosen for its relevance to minimally–speaking adults – we selected topics that autistic adults themselves often reference as central to their communication successes and failures. Throughout all sections, we note specific samples in included studies, a practice we encourage other researchers to adopt.

## Literal and Non-Literal Language

Language is flexible; the meaning of a word often changes depending on the situation [18, 19]. For example, the word *dish* can mean a plate, a meal, the act of serving food, or even gossiping. About 80% of English words have multiple related meanings. This phenomenon makes communication flexible, but speakers and writers must share enough context for listeners and readers to pinpoint the intended meaning in the moment [20].

Many people assume that words have simple, fixed meanings, but even the most common words represent complex and nuanced ideas shaped by context [18, 19, 21]. The same holds true for phrases. Consider the expression *spill the beans,* which means “to reveal a secret,” rather than literally dropping beans. The ability to discern context is particularly challenging for autistic people. Importantly, this difficulty persists even when controlling for general language ability [22].

When matched on age, education, or performance tasks, autistic adults perform comparably to or better than non-autistic adults on structured language tasks, including memorizing new words [23], context-independent vocabulary tests [24], and overall lexical knowledge [22]. However, when tasks require extending word meanings in novel ways [23], making normative judgments on new stimuli [24], interpreting implied or non-literal meanings [24, 25], or integrating thematic elements (e.g., in storytelling; 27), autistic adults tend to struggle.

These patterns suggest that autistic adults often excel in structured, rule-based language tasks, but face greater challenges in more flexible, interpretive contexts that rely on extending or abstracting meaning. One notable factor underlying these challenges is a preference for literal interpretations of language (for a review, see 30). This tendency presents challenges in tasks like metaphor processing, which requires understanding non-literal language. Such difficulties are frequently linked to broader language impairments; verbal ability is a key predictor of success in metaphor comprehension (for a review, see 29). Literal interpretation can create miscommunication, and autistic adults may compensate by relying heavily on memory. One autistic individual shared, *"I wish people were more mindful of things that are ambiguous in communication. I’m fortunate to have memorized so many idioms and metaphors that I can instantly translate them in my head"* [26].

Despite these challenges, a preference for literal interpretation has advantages in specific contexts, including technical or legal writing, where clarity and precision are critical. Autistic individuals may also demonstrate less conventional but highly effective language use, characterized by precision and creativity [27].

## Augmentative and Alternative Communication (AAC)

**Fig. 1:**
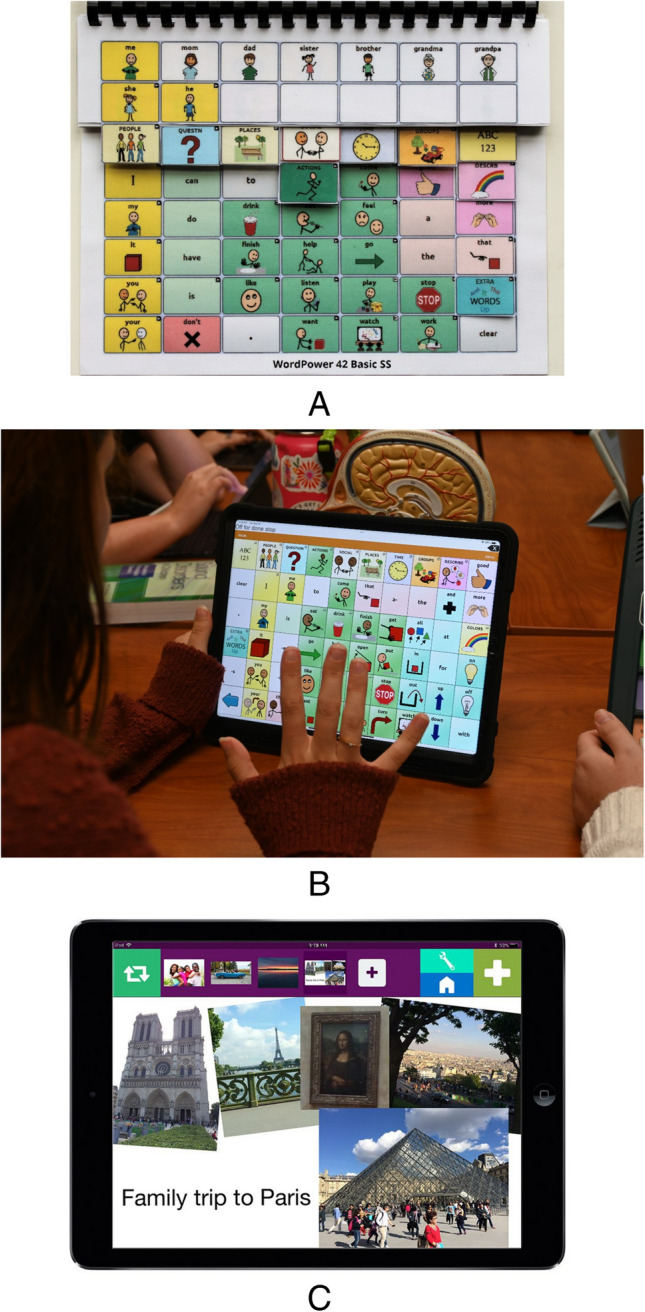
Examples of low- and high-tech AAC displays.** a** Example of a lite/low-tech paper-based AAC option using both orthographic and picture symbols to support communication. Photo courtesy of PRC-Saltillo [43]. Used with permission. **b** Example of high-tech AAC option, Touchchat mobile application, on an iOS device. Photo courtesy of Salus at Drexel University, Speech-Language Pathology Program. **c** Example of Just in Time programming using a Visual Scene Display. Contextual personal photographs are uploaded to the GoVisual application [44]. Photos contain hotspots programmed with messages for communicating with others about a recent family trip. GoVisual is an Attainment Company Software Publication; image used with permission

Augmentative and alternative communication (AAC) refers to tools and strategies that support individuals with complex communication needs. These range from low-tech options such as paper based, picture communication (Fig. 1A) to high-tech solutions such as speech-generating devices (29, 30; Fig. 1B). AAC systems enhance communication, foster independence, and improve quality of life by supporting the AAC user in important, everyday life activities [28]. The studies reviewed in this section primarily include minimally speaking adults.

For autistic adolescents and adults, AAC access improves functional outcomes, social communication, and engagement [29]. AAC positively impacts employment outcomes for non-speaking autistic adults by enhancing employee and employer satisfaction [30–32]. For autistic adults with significant communication challenges, AAC tools are essential for social interaction and self-expression [33]. However, most existing literature focuses on AAC use in children. This gap highlights the need to better understand AAC’s role across the lifespan, particularly in supporting autistic adults [28, 34].

Recent advancements in AAC technology, such as just-in-time programming and visual scene displays (Fig. 1C), are transforming communication for individuals with high communication needs. These tools enable users to instantly capture real-world scenes and access context-specific vocabulary [35–38]. Innovations like AI-generated vocabulary from photographs further enables devices’ ability to meet users’ ‘in-the-moment’ communication needs [36, 39]. These technologies support participation in complex social situations and address sensory and anxiety-related difficulties that arise when verbal speech is inaccessible [40, 41].

Successful AAC implementation requires careful planning, training, and support from communication partners, including family members, friends, community members, educators, and employers [28]. Individuals familiar with symbolic communication adapt more easily to this role, while AAC newcomers require more intensive training. Training models like the Communication Partner Instruction framework equip partners with the skills to integrate AAC use to everyday contexts, improving communication for users with severe disabilities [42].

The continued evolution of AAC technology requires ongoing research to ensure these goals and strategies remain effective, accessible, and adaptable to autistic people’s wide-ranging communication needs. AAC tools and systems not only enable communication but also promote inclusion, independence, and opportunities for meaningful engagement.

## Nonverbal Communication

Nonverbal communication (sometimes referred to as “multimodal language”) refers to the integration of the many modalities of our communicative system: we can speak (the vocal modality), but we also use facial expressions, manual gestures, and posture (the visual modality). Not everyone uses all possible signals or modalities all the time (see also the section on AAC), but nonverbal communication is ubiquitous, particularly in face-to-face communication. We note that all studies in this section include only verbally fluent autistic adults, due to limitations in the available research literature.

Nonverbal communication serves a wide range of functions. Different modalities are often co-expressive: information conveyed via language may be redundant with other modalities or communicate entirely new information [45]. Often, interpreting meaning requires *integrating* multiple modalities. Consider the exclamation, “look!” Its meaning may depend on *tone* – is it spoken in awe or alarm? – or on whether the speaker is gesturing toward a cardinal hiding in a tree or a car running a red light. Nonverbal communication thus links speech and the environment. As such, it often plays a critical role in revealing speakers’ intentions [46]. Nonverbal communication also serves interactive functions such as grammatical marking, emphasis, and regulation of conversational turn-taking [47].

Several studies now demonstrate that autistic adults benefit from nonverbal communication similarly to how non-autistic adults do [48, 49]. For example, when speech is hard to hear, gestures can support comprehension [50, 51], an especially important finding given that auditory barriers are reported as highly influential on autistic adults’ ability to participate in conversations [52–54]. Recent studies show little to no evidence for overall group differences in facial emotion recognition accuracy [55–58]; however, alexithymia (i.e., difficulty expressing, experiencing, or identifying emotions) traits seem to modulate the intensity [57] and accuracy of perceived emotions [59]. These findings show the importance of considering how co-occurring conditions can shape traits related to the traditional autism phenotype.

Among speaking autistic adults, differences in nonverbal communication relate to differences in processing or executing contextual cues, a pattern observed across behavioral [49] and neuroimaging data (gesture: [50, 60]; facial expressions: [59], prosody: [61]), and autistic adults’ own descriptions of their experiences [62]. Importantly, even when there are no differences behaviorally, neuroimaging studies suggest that autistic individuals may have a different way of *processing* information than non-autistic adults [50, 60]. These processing differences could impact the interpretation of others’ nonverbal behaviors, or the time or effort required to do so.

The nonverbals autistic people *produce* also differ from control groups, for example, their gestures are more likely to be unilateral [63] and to show greater temporal segmentation through the use of brief pauses in movement [49]. Such differences may impact the way others interpret autistic individuals’ nonverbal cues. Autistic adults appear to rely heavily on nonverbal communication (specifically, hand gestures) to support conversational turn-taking, supporting social benefits to autistic speakers [63]. In the vocal modality, the backchannels (e.g., ‘ums’ or ‘uhs’ within conversational pauses that cue states such as confusion, attention, and needing more time) that autistic adults produce during conversation may also differ prosodically [64]. This likely impacts mutual understanding.

Autistic adults are aware of the different ways they use and understand nonverbal communication, and the negative impacts these differences can have on their lives. For example, autistic adults describe negative impacts when others assume they are being dishonest due to their facial expression not “matching” their verbal language. Autistic adults also describe how it may be easy to interpret emotional extremes, but challenging to interpret more subtle expressions [65]. Many autistic adults describe how their greatest challenges with multimodality involve processing differences such as needing more time to interpret and/or integrate multiple signals. One contributor to Radford and colleagues’ study [62] stated, “*it's not so much a case of ‘can/cannot’ read body language, so much as a different way of doing it which has a much higher cognitive load, so is much more tiring.*”

## Double Empathy

Traditional autism literature focuses on social and cognitive difficulties in the autistic child or adult. Accordingly, it often concludes that observed interaction difficulties arise due to the autistic person’s deficits [66, 67]. A newer theory, the “Double Empathy Problem” (DEP), emphasizes the bidirectional nature of forming and maintaining social relationships. [68]. Originally described and developed in large part by autistic scholars, the DEP shows how interactions between autistic and non-autistic partners sometimes break down because non-autistic people may not understand different communication styles, akin to cultural differences that can hinder mutual understanding.

Empirical evidence supporting this theory has accumulated over more than a decade of study [69]. For example, non-autistic people struggle to understand the mental states of autistic people [70] which might lead them to less favorable impressions [71]. Social dynamics often associated with autism (e.g., low rapport) improve when measured during interactions exclusively among autistic people [72], as behavioral indicators of rapport differ between autistic and non-autistic pairs. While mutual gaze and backchanneling lead to higher rapport in non-autistic pairs, *less* use of these signals predicts higher rapport in autistic pairs [73], demonstrating how a difference – but not a deficit – in nonverbal communication can hinder social connectedness across – but not within – neurotypes.

The DEP emphasizes differences in autistic culture that may drive more positive interactions between autistic people, relative to across neurotypes. For example, among autistic people, it is often considered polite to ask questions of a communication partner to confirm mutual understanding. However, non-autistic people often misinterpret this same behavior as rude, difficult, dominant, or challenging authority. Non-autistic people could learn to respect autistic social norms/etiquette, rather than placing the sole burden on autistic people to conform to non-autistic expectations.

As described above, autistic adults are less likely to follow neurotypical language conventions [53, 54, 74], sometimes resulting in misunderstandings. Misunderstandings due to differences in communication and interaction styles across neurotypes have implications for autistic people in critical life domains, including education [75], employment [76], and healthcare [77]. The shift in focus from autistic deficits to neurodivergent misalignment is essential for advancing our understanding of contextual communication. It suggests that non-autistic people can improve communication with autistic people by adapting their own expectations and behaviors. Below, we make suggestions for those who are looking to do just that.

## Recommendations

Autistic adults are encouraged to learn self-advocacy skills to communicate their needs and preferences [78]. It is equally important that non-autistic people, including teachers, parents, carers, and clinicians, advocate for the rights and needs of neurodivergent people and learn skills themselves to promote and support autistic communication. Given the many barriers neurodivergent people face and the danger of self-advocacy fatigue or burnout, neurodivergent people need allies [79, 80]. Psychiatrists can be allies by educating themselves and their colleagues about autistic communication differences and how to support those differences to enhance mutual understanding.

### Clinical Implications and Recommendations

Autistic adults experience communication difficulties as arising from both internal and external factors, and suggest that communication supports should focus on building skills for *both* autistic people and society at large [53]. For example, difficulty communicating with providers is a barrier to healthcare for autistic adults [81], who have significantly shorter lifespans compared to the general population [82]. Autistic patients identify both their own communication skills and their *providers’* communication skills (e.g., use of accessible language) as major factors influencing healthcare interactions [77, 81]. With respect to mental health specifically, Maddox and colleagues [83] investigated community mental health services for autistic adults, identifying barriers at the clinician, client, and systemic levels. They found that clinicians lack training and confidence in working with autistic clients, while communication differences make it difficult for autistic adults to express their needs or navigate the healthcare system. Systemic issues, such as poor coordination between mental health and disability services, often result in denied care or referral to inappropriate services [83].

In addition to needed systemic changes, individual clinicians can improve their patients’ health outcomes by adjusting their own communication practices. Table 1 includes examples of specific communication differences often observed in or reported by autistic people, with accompanying recommendations for clinicians on how to manage them. Critically, communication differences vary widely among autistic and neurodivergent people in general. Thus, clinicians who want to be better communication partners with their autistic and neurodivergent patients should make fewer assumptions about what these patients can and cannot do, while also self-educating about common communication differences to be on the lookout for.

**Table 1:** Common communication differences reported by or observed in autistic adults, and recommendations for clinician adaptations

**GOAL: LOWER COGNITIVE AND EMOTIONAL DEMANDS**	
**Communication Difference**	**Processing and response time**
* Example(s)*	*• Patient responds more slowly than expected*
* Clinical adaptations (general)*	*• Slow down speaking pace and use more concise language when possible (fewer words to process and more time to do so)* *• Allow time for processing without assuming disengagement* *• Minimize small talk, which can be draining, and jump straight to the point of the session/appointment* *• Reduce expectations of immediate backchanneling (e.g., nodding, saying “uh huh” while listening/thinking)*
* Adaptation examples and scripts*	*• Count to ten before prompting for a response * *• Allow longer pauses instead of quickly filling silence* *• Instead of rushing to fill a silence, consider all of the internal work your patient may be doing in their own mind before they are ready to move forward. Consider acknowledging the work that happens during silence* *• Explicitly allow an opportunity for the patient to ask questions or add comment before moving onto another subject, “Do you have any other questions before we talk about something else?”*
**Communication Difference**	**Cognitive/emotional overload**
* Example(s)*	*• Patient becomes overwhelmed when asked multiple complex questions* *• Patient-clinician communication goes well, but patient fatigues quickly*
* Clinical adaptations (general)*	*• Use a predictable and consistent structure during appointments, so that patient knows what to expect* *• Send questions and session agenda in advance so that patient is able to prepare* *• Pace the session by asking one question at a time, allowing the patient to fully answer before moving on* *• Offer pauses or breaks if signs of overload are endorsed or observed* *• May need to offer multiple, shorter appointments to minimize fatigue*
* Adaptation examples and scripts*	*• “First, I’d like to hear about anything you need to tell me since we last met, and then I will ask you about medication side effects.”* *• “Once you’re ready, we can talk more about what happened.”* *• Provide a written summary of the discussion and clear action points for care plan, as it may be difficult for the patient to maintain conversation while also retaining information about their care plan. This can happen during the last few minutes of a session, simultaneously serving as a time the patient can be still and regulate before leaving the session*
**GOAL: REDUCE BIAS AND ASSUMPTIONS**	
**Communication Difference**	**Differences in nonverbal expression (e.g., eye contact, facial expression, vocal tone)**
* Example(s)*	*• Patient looks away, makes minimal eye contact, or has a neutral facial expression* *• Patient’s facial expressions do not “match” expected emotional cues based on their verbal language*
* Clinical adaptations (general)*	*• Recognize multiple valid ways of expressing engagement* *• Focus on explicit communication over inferred meaning* *• Avoid assuming a patient is not paying attention if they are fidgeting or stimming; these behaviors can often improve regulation and focus* *• Use verbal check-ins rather than relying on assumed meanings of nonverbal expressions*
* Adaptation examples and scripts*	*• Instead of assuming disengagement, focus on what the patient is saying* *• Listen to what your patient is saying, not how they are behaving* *• Instead of inferring patient’s emotional state based on nonverbal cues, ask: “How do you feel about that?” or “How are you feeling right now?”*
**Communication Difference**	**Differences in understanding implicit social expectations**
* Example(s)*	*• Clinician is uncertain about their patient’s understanding of session/appointment expectations*
* Clinical adaptations (general)*	*• Check in about communication preferences instead of making assumptions* *• Explicitly confirm understanding and preferences (make the implicit explicit)* *• Consider explicitly acknowledging that you understand and welcome common nonverbal differences, such as reduced eye contact, long pauses, fidgeting/stimming* *• Check in to ensure what you have communicated is understood and you interpreted the patient correctly*
* Adaptation examples and scripts*	*• “Is my speaking pace okay for you?” or “Would you like me to slow down?”* *• "Is this a correct interpretation of what you just told me?"* *• “I want to make sure I’ve explained everything clearly…”*
**GOAL: ACCOMMODATE LANGUAGE PREFERENCES**	
**Communication Difference**	**Preference for precise, literal language**
* Example(s)*	*• Patient interprets figurative language (e.g., “Let’s unpack this”; “Where are we on that goal?”) literally (e.g., shows confusion about what to unpack or gives a physical location in response to a question intended to be about progress toward goals)*
* Clinical adaptations (general)*	*• Use direct and concrete language* *• Be on the lookout for idioms and metaphors that are part of your automatic communication style* *• Listen with a literal ear: be on the lookout for times when you may be interpreting your patient’s statements in a non-literal way, when they may be intended as literal statements* *• Rephrase statements in a way that prioritizes clarity*
* Adaptation examples and scripts*	*• Instead of “Let’s unpack this,” say “Can you describe that in more detail?”*
**Communication Difference**	**Preference for alternative communication methods**
* Example(s)*	*• Patient prefers writing to speaking*
* Clinical adaptations (general)*	*• Offer multiple ways to communicate, including visuals like drawing and using emojis* *• Allow for written, typed, or asynchronous communication options*
* Adaptation examples and scripts*	*• “You can always email me after the session if there’s something else you’d like me to know. You can also email me any questions you have at any time.”*

In addition to general adaptations and communication practices, there is now a growing evidence base for mental health interventions that have been tailored to and evaluated with autistic patients. Increasingly, autistic researchers and community partners have helped develop or adapt these programs, reflecting best practices. As one example, take the problem of suicide. Autistic individuals have a heightened risk of suicide: recent studies suggest a two- to eight-fold increase compared to the general population. Prevalence studies estimate that 34% of autistic individuals experience suicidal ideation, with 24% reporting suicide attempts​ [84]. The CBT-DAY model, a co-created neurodiversity-affirming group intervention, has demonstrated effectiveness in reducing depressive symptoms and targeting key factors linked to suicidal behaviors in autistic adults. Scaling these interventions and reforming systemic shortcomings are critical to mitigating the heightened suicide risk faced by autistic people and improving their mental health outcomes [85, 86].

Lastly, an autistic person cannot be fully understood in isolation from other constructs such as their race, gender, sexuality, class, etc. These multiplying identities create diverse experiences and can create additional barriers to interaction [87]. Again, we advocate for personalized care based on each individual patient’s identities and referring problems. Overall, anticipating and responding to – not only understanding – the diverse experiences and needs of autistic people is the best way to ensure they thrive in society, education, and employment.

### Recommendations for Research and Researchers

As recommended to clinicians, we also encourage researchers to anticipate communication differences and pay attention to how their own communication styles and preferences may create barriers to communicating with autistic research participants. As described in the introduction, research in general is limited in autistic adult samples, thus we strongly encourage researchers and funders to increase their research portfolios to focus on adults in addition to children. Some progress has been made on research showing how non-autistic adults can change their own communication practices to be better interaction partners for autistic people [88]; this is a critical research area in need of more work.

As much current research tends to focus on autistic samples with higher IQs and lower support needs, we recommend increasing inclusivity and access to research for autistic adults with higher support needs, especially those who may be non-speaking or minimally-speaking. In our own work, for example, we have implemented a range of strategies to make participation accessible to as many people as possible. Participants may complete our studies independently, with a trusted support person, with support from our study team, or, in the case of adults who cannot self-report, we allow participation by proxy. Occasionally, ethical dilemmas present themselves while trying to enhance fair subject selection and inclusivity while balancing rigorous data collection. We recently discussed some of these tradeoffs with respect to online data collection with autistic adults [89].

Communication is a research priority for autistic adults. At a 2019 summit, the Autistic Self-Advocacy Network identified the question, “how can we help autistic people communicate?” as a key research concern [14]. Autistic adults advocate for research that characterizes autistic communication and improves accessibility, yet most funding remains concentrated on biomedical and genetic studies rather than everyday communication solutions that respect autistic agency [90, 91]. Additionally, autistic adults want a greater investment in AAC, nonverbal communication interventions, and accommodations for sensory and cognitive differences [92, 93]. Participatory approaches and the removal of research barriers, including exclusionary criteria, deficit-based models, and inaccessible methods, are necessary to align autism research with autistic priorities and improve communication support [14, 77, 94].

We support efforts to include autistic adults, not only as research participants, but as part of the research team. Doing so ensures both that research questions are relevant to the autism community, and that research practices are as accessible as possible. For example, AASPIRE, a longstanding community academic collaborative, has published guidelines on how to engage autistic community partners as co-researchers [95], and guidelines for conducting accessible research [96].

## Conclusions

Research on autistic adults, especially non- and minimally-speaking populations, is limited and requires focused effort. Improved communication is associated with improved outcomes including autonomy, social relationships, and purpose, at all IQ levels. Critically, because communication is bilateral, improving communication for autistic adults requires adaptation and change by non-autistic communication partners. Important but understudied areas of research and clinical practice include communication partner training for both speaking [97] and minimally-speaking adults [42]. Clinicians should prioritize understanding communication differences and nonverbal cues in their interactions, while minimizing assumptions that may be based on non-autistic behaviors, or assumptions about a patient just because they are autistic. Allyship, intersectionality, and understanding autism as a culture will support impactful research and practice. Psychiatrists can be allies not only by supporting their own patients but also by educating colleagues and the next generation of clinicians.

## Key References

- Sterrett K, Clarke E, Nofer J, Piven J, Lord C. Toward a functional classification for autism in adulthood. Autism Res. 2024;17(10):2105–19.Sterrett K, Clarke E, Nofer J, Piven J, Lord C. Toward a functional classification for autism in adulthood. Autism Res. 2024;17(10):2105–19.
  - This study describes the support needs of a longstanding longitudinal cohort of autistic participants at aroundage 30. Support needs were determined via detailed interviews with autistic participants or a caregiver, focusing on the proband's daily life. The authors created a structured decision tree to categorize support needs.
- Cummins C, Pellicano E, Crane L. Autistic adults’ views of their communication skills and needs. Int J Lang Commun Disord. 2020;55(5):678–89.
  - Qualitative study focused on how (speaking) autistic adults experience communication. An excellent source for examples of communication differences, in the words of autistic people themselves.
- Luyster RJ, Zane E, Wisman WL. Conventions for unconventional language: Revisiting a framework for spoken language features in autism. Autism Dev Lang Impair. 2022;7:239694152211054.
  - A helpful overview of common language atypicalities observed in autism, and how they both relate, and do not relate, to communication.
- Milton D, Gurbuz E, López B. The ‘double empathy problem’: Ten years on. Autism. 2022;26(8):1901–3.
  - 10-year update on the “double empathy” hypothesis.

## Data Availability

No datasets were generated or analysed during the current study.

## Footnotes

1 Terminology used to describe autism and associated ability/disability is a hotly debated topic. Because there is heterogeneity in terms of how adults on the autism spectrum preferred to be referred (1), we use a range of terminology in this manuscript. In our empirical work, we ask our autistic participants for their preferred terminology, and use the most common selections in our publications (2). Our guiding philosophy for thinking and writing about autistic communication is to not make assumptions that differences are necessarily deficits – although sometimes they can be (3). For guidance on avoiding ableist language and pathologizing frameworks, please see (1). We briefly note our own positionality: **AdM** is a non-autistic licensed clinical psychologist and autism researcher. She enjoys many close relationships with autistic adults, and caregivers of autistic adults, primarily through research collaborations and participatory methods. **NC** is a non-autistic researcher and ally investigating communication differences to promote better language practices and enhance accessibility guidelines. **EG** is a non-autistic autism researcher and autism ally working with autistic community to understand support needs in higher education and employment. **MB** is a neurodivergent, gender diverse autism researcher in training committed to research that prioritizes autistic lived expertise. **JT** is an autistic communication researcher. He tries to understand communication through the lens of neurodiversity, and engages with autistic researchers and advisory groups to better situate his research. **JB** is a SLP and AAC specialist working within autistic community by supporting effective communication strategies across the lifespan.
